# Effect of the *PPARG2* Pro12Ala Polymorphism and Clinical Risk Factors for Diabetes Mellitus on HbA1c in the Japanese General Population

**DOI:** 10.2188/jea.JE20120078

**Published:** 2012-11-05

**Authors:** Megumi Hara, Yasuki Higaki, Naoto Taguchi, Koichi Shinchi, Emi Morita, Mariko Naito, Nobuyuki Hamajima, Naoyuki Takashima, Sadao Suzuki, Akihiko Nakamura, Keizo Ohnaka, Hirokazu Uemura, Hideki Nishida, Satoyo Hosono, Haruo Mikami, Michiaki Kubo, Hideo Tanaka

**Affiliations:** 1 Department of Preventive Medicine, Faculty of Medicine, Saga University, Saga, Japan; 1 佐賀大学医学部社会医学講座予防医学分野; 2 Laboratory of Exercise Physiology, Faculty of Sports and Health Science, Fukuoka University, Fukuoka, Japan; 2 福岡大学スポーツ科学部運動生理学研究室; 3 Division of International Health and Nursing, Faculty of Medicine, Saga University, Saga, Japan; 3 佐賀大学大学院医学系研究科地域・国際保健看護学講座; 4 Department of Preventive Medicine, Nagoya University Graduate School of Medicine, Nagoya, Japan; 4 名古屋大学大学院医学系研究科予防医学; 5 Department of Health Science, Shiga University of Medical Science, Otsu, Japan; 5 滋賀医科大学社会医学講座公衆衛生学; 6 Department of Public Health, Nagoya City University Graduate School of Medical Sciences, Nagoya, Japan; 6 名古屋市立大学大学院医学研究科公衆衛生学分野; 7 Department of International Island and Community Medicine, Kagoshima University Graduate School of Medical and Dental Science, Kagoshima, Japan; 7 鹿児島大学大学院医歯学総合研究科国際島嶼医療学講座; 8 Department of Geriatric Medicine, Kyushu University Graduate School of Medical Sciences, Fukuoka, Japan; 8 九州大学大学院医学研究院老年医学分野; 9 Department of Preventive Medicine, Institute of Health Biosciences, University of Tokushima Graduate School, Tokushima, Japan; 9 徳島大学大学院ヘルスバイオサイエンス研究部予防医学; 10 Department of Epidemiology for Community Health and Medicine, Kyoto Prefectural University of Medicine, Kyoto, Japan; 10 京都府立医科大学大学院医学研究科地域保健医療疫学; 11 Division of Epidemiology and Prevention, Aichi Cancer Center Research Institute, Nagoya, Japan; 11 愛知県がんセンター研究所疫学・予防部; 12 Division of Cancer Registry, Prevention and Epidemiology, Chiba Cancer Center, Chiba, Japan; 12 千葉県がんセンターがん登録・予防疫学研究室; 13 Laboratory for Genotyping Development, Center for Genomic Medicine, RIKEN, Yokohama, Japan; 13 理科学研究所ゲノム医科学研究センター

**Keywords:** peroxisome proliferator-activated receptor-γ2, polymorphism, glycated hemoglobin, interaction

## Abstract

**Background:**

Although the peroxisome proliferator-activated receptor-γ2 (*PPARG2*) Pro12Ala gene variant is associated with diabetes mellitus, the associations and interactions of this polymorphism and known clinical risk factors with glycated hemoglobin (HbA1c) remain poorly understood. We investigated if carrying the Ala allele was inversely associated with HbA1c level and examined possible interactions.

**Methods:**

This cross-sectional analysis used data collected from 1281 men and 1356 women aged 40 to 69 years who completed the baseline survey of the Japan Multi-Institutional Collaborative Cohort Study. *PPARG2* polymorphism was determined by multiplex polymerase chain reaction (PCR)-based Invader assay. Multiple linear regression and ANCOVA were used to control for confounding variables (age, body mass index [BMI], energy intake, alcohol, smoking, physical activity, and family history of diabetes) and examine possible interactions.

**Results:**

After adjustment, the Ala allele was significantly inversely associated with HbA1c in women but not in men. Older age, BMI, and family history of diabetes were associated with higher HbA1c in both sexes. When stratified by *PPARG2* genotype, these associations were observed in subjects with the Pro12Pro genotype but not in Ala allele carriers. A significant interaction of genotype and BMI on HbA1c was observed in women. Older age, BMI, and family history of diabetes were significantly associated with high-normal HbA1c (≥5.7% NGSP), whereas *PPARG2* polymorphism was not.

**Conclusions:**

Although *PPARG2* Pro12Ala polymorphism might attenuate associations between known risk factors and HbA1c level, it had a small effect on high-normal HbA1c, as compared with clinical risk factors, in the general population.

## Abstract (ja)

**背景：:**

肥満や脂質代謝に関連する遺伝子の*PPARG2*の変異型の人で糖尿病のリスクが低いことが分かってきたが、糖尿病の危険因子と*PPARG2*の遺伝子型がHbA1cに与える影響は十分に検証されていない。そこで、*PPARG2*の遺伝子の型と糖尿病の危険因子がHbA1c値に及ぼす影響、および交互作用について検討した。

**方法：:**

J-MICC Studyのベースライン調査に参加した40～69歳の男性1281人、女性1356人のデータを用いて横断研究を実施した。遺伝子多型はMultiplex PCR-based Invader assay方法により決定した。男女別に、Pro12Ala多型とHbA1cの関連について、開始時の年齢、エネルギー摂取量、身体活動量、飲酒状況、喫煙状況、BMIで調整し、共分散分析、重回帰分析を行った。

**結果：:**

女性では、*PPARG2*遺伝子のPro12Ala型及びAla12Ala型を持つ者はPro12Pro型を持つ者と比較して、HbA1cが有意に低かった。*PPARG2*がPro/Pro型の人は、加齢や肥満、家族歴があるとHbA1cの上昇がみられたが、Pro/AlaまたはAla/Ala型の人ではみられなかった。女性ではHbA1c値に対しBMIと遺伝子多型による有意な交互作用がみられた。加齢や肥満、家族歴はHbA1c高値と有意な関連がみられたが、*PPARG2*遺伝子の多型とHbA1c高値の間に有意な関連はみられなかった。

**結論：:**

*PPARG2*遺伝子のPro12Ala多型は、HbA1cと既知の危険因子の関連に影響を与える可能性があるが、一般集団においてHbA1c高値に対する影響は既知の危険因子に比べて小さかった。

## INTRODUCTION

The prevalence of type 2 diabetes mellitus (type 2 diabetes) has markedly increased during the last decade. The number of people living with diabetes is expected to rise from 366 million in 2011 to 552 million by 2030 if no urgent action is taken.^1^^,^^2^ According to the 2010 National Health and Nutrition Survey, about 17.4% of Japanese men and 9.6% of Japanese women may have diabetes.^3^ Because age is an important risk factor for type 2 diabetes, the number of patients will further increase in Japan, which will result in a serious public health problem.

Type 2 diabetes is a complex of lifestyle-related clinical risk factors^4^ and genetic factors.^5^ Among clinical risk factors, increased body mass index (BMI), excessive energy intake, physical inactivity, and smoking have been associated with a higher risk of diabetes, while moderate alcohol use has been associated with a lower risk.^4^ Among genetic factors, family history has a large effect on predicting the development of type 2 diabetes.^4^^,^^6^

During the last decade, a number of genetic variants have been examined for their association with type 2 diabetes, and a consistent association has been found for peroxisome proliferator-activated receptor-γ2 (*PPARG2*).^7^ A recent meta-analysis reported that the *PPARG2* Pro12Ala polymorphism was associated with a reduction in type 2 diabetes risk and that this association did not differ between Asians and whites.^7^ However, the interactions between this polymorphism and known clinical risk factors (including family history of diabetes) for type 2 diabetes risk are not well understood.

Glycated hemoglobin (HbA1c) is commonly used to diagnose diabetes and can also be used to identify individuals at higher risk of developing diabetes. In 2010, the American Diabetes Association suggested that prevention strategies should be particularly intensive among people with a high-normal HbA1c level (5.7%–6.4%, National Glycohemoglobin Standardization Program [NGSP] values), because this population has the greatest risk of developing diabetes.^8^ Recently, Heianza et al reported that the predictive value of a high-normal HbA1c for diabetes progression was similar to of impaired fasting glucose alone in the Japanese population.^9^ To identify a genetic factor other than family history that is associated with high-normal HbA1c would be useful for implementing early-prevention strategies against diabetes.

The purpose of this study was to examine if carrying the Ala allele of *PPARG2* was inversely associated with HbA1c and if this association modified the effects of known clinical risk factors, family history of diabetes, and their interactions. In addition, we investigated whether *PPARG2* polymorphism was associated with high-normal HbA1c after adjusting for possible confounders.

## METHODS

### Study participants

The Japan Multi-Institutional Collaborative Cohort (J-MICC) Study is a large genome cohort followed to confirm and detect gene–environment interactions in lifestyle-related diseases. The details of the cohort have been described elsewhere.^10^ Briefly, the J-MICC Study was initiated 2005, and participants aged 35 to 69 years were enrolled voluntarily from 10 areas of Japan. In the present cross-sectional study, we used data from 4519 participants enrolled throughout Japan during 2004–2008.^11^ Written informed consent was obtained from all participants, and the study protocol was approved by the ethics committees of Nagoya University School of Medicine and the participating institutions.

### Questionnaire and measurements

A self-administered questionnaire including items on alcohol consumption, smoking, dietary habits, current medication, past disease history, and first-degree family history of diabetes was used for data collection. For dietary assessment, a validated food-frequency questionnaire (FFQ) was used, and intakes of energy, fat, protein, carbohydrates, and ethanol were calculated.^12^^–^^14^

Physical activity was assessed in terms of metabolic equivalents (METs) of daily and leisure-time activity. MET values less than 3 were not counted as physical activity. Participants reported the average time per day spent doing heavy physical work (assigned MET intensity: 4.5 METs), walking (3.0 METs), standing (<3.0 METs, not counted as physical activity), and engaged in sedentary activity (<3.0 METs, not counted as physical activity). Response options were as follows (assigned average time per day in parentheses): none (0), less than 1 hour/day (0.5), 1 to less than 3 hours/day (2.0), 3 to less than 5 hours/day (4.0), 5 to less than 7 hours/day (6.0), 7 to less than 9 hours/day (8.0), 9 to less than 11 hours/day (10.0), and 11 or more hours/day (12.0). MET-hours per day (MET·h/day) of daily activity was estimated for heavy physical work and walking. For leisure-time activity, participants were asked about the frequency and average duration of low-intensity exercise (3.4 METs), moderate-intensity exercise (7.0 METs), and high-intensity level exercise (10 METs). The frequency categories (assigned daily average frequencies in parentheses) for leisure-time activity were almost none (0), 1 to 3 times/month (0.1), 1 to 2 times/week (0.2), 3 to 4 times/week (0.5), and 5 to 6 times/week (0.8). The categories for average duration (assigned average hours per activity in parenthesis) were less than 30 minutes (0.3), 30 minutes to less than 1 hour (0.8), 1 to less than 2 hours (1.5), 2 to less than 3 hours (2.5), 3 to less than 4 hours (3.5) and 4 or more hours (4.5). MET·h/day of leisure-time activity was estimated by multiplying the reported daily time spent in each activity by the assigned MET intensity. After summing across daily and leisure-time activity, participants were divided into 4 groups by quartile of MET·h/day and stratified by sex.

Height and weight were measured to the nearest 0.1 cm and 0.1 kg, respectively. HbA1c (%) was measured in laboratories in each study area, and the results of these measurements were collected. The equation for conversion from HbA1c (Japan Diabetes Society [JDS]) to HbA1c (NGSP) units is officially certified as follows: NGSP(%) = 1.02 × JDS(%) + 0.25%.^15^ According to Heianza et al,^9^ the sum of the sensitivity and specificity for identifying individuals with impaired fasting glucose among those with an HbA1c ranging from 5.7% to 6.4% was highest when HbA1c was 5.7%, so we used 5.7% as the HbA1c cut-off to define high-normal HbA1c.

### Genotyping of polymorphism

Genotyping details have been described elsewhere.^11^ Briefly, 107 single-nucleotide polymorphisms, including the *PPARG2* Pro12Ala gene (rs1801282), were genotyped using a multiplex polymerase chain reaction (PCR)-based Invader assay (Third Wave Technologies, Madison, WI, USA)^16^ at the Laboratory for Genotyping Development, Center for Genomic Medicine, RIKEN. Genotype distributions were tested for Hardy–Weinberg equilibrium, and the *P* value (on the exact test) for the *PPARG2* Pro12Ala gene was 0.80.^11^ After genotyping, data from 6 participants who withdrew from follow-up were excluded from further analysis.

### Statistical analysis

In the analysis, we excluded 1877 participants who were missing data on *PPARG2* polymorphism (*n* = 6) or HbA1c (*n* = 1768), were on type 2 diabetes medication (*n* = 193), or had a dietary energy intake greater than 4000 kcal/day (*n* = 2). Consequently, data from 1280 men and 1356 women aged 40 to 69 years were included in the analysis. Among these participants, some were missing data on alcohol consumption (20 men and 22 women), BMI (1 man), or physical activity (9 men and 14 women).

All analyses were performed with the SAS statistical software package (Ver. 9.1 for Windows; SAS Institute, Cary, NC, USA). A *P* value of less than 0.05 was considered statistically significant. Intakes of total energy, fat, protein, and carbohydrates were estimated by the SAS software using the information from the FFQ and standard tables of food composition in Japan (Fifth Revised Edition).^17^ The SAS software and FFQ were developed by the same researchers.^12^^,^^13^ For comparison of participant characteristics by sex we used the *t* test (for continuous variables) and the χ^2^ test (for categorical variables). Crude and adjusted mean HbA1c values and their 95% CIs were evaluated by least-squares general linear regression, and linear trends were assessed by the statistical significance of the regression coefficient of an ordinal variable for the factor under consideration as follows: age category (35–39, 40–49, 50–59, or 60–69 years), BMI quartile, energy intake quartile, fat intake quartile, protein intake quartile, carbohydrate intake quartile, physical activity quartile, alcohol consumption status (never, former, or current drinker consuming 0.1–22.9, 23.0–45.9, or ≥46.0 grams ethanol/day), smoking status (never, former, or current smoker of 1–19, 20–39, or ≥40 cigarettes/day), first-degree family history of diabetes (positive, negative, or unknown), and *PPARG2* genotype (Pro12Pro, Pro12Ala, or Ala12Ala). Regarding family history of diabetes, we did not use data from participants with an unknown family history in the tests for trend. To further explore the effects of *PPARG2* genotype and clinical risk factors on HbA1c levels we divided each clinical risk factor into 2 groups as follows: age (35–49 or 50–69 years), BMI (<23.6 or ≥23.6 kg/m^2^ for men and <22.4 or ≥22.4 kg/m^2^ for women), energy intake (<1880 or ≥1880 kcal/day for men and <1540 or ≥1540 kcal/day for women), alcohol consumption status (never, former, or current drinker), and family history (positive or negative). *PPARG2* genotype was also divided into 2 groups (Pro12Pro or Ala allele carrier). Because of the low frequency or absence of minor homozygous participants within these groups, they were combined with heterozygous participants. Crude and adjusted mean HbA1c values and their 95% CIs and linear trends were computed with respect to genotype and clinical risk factors. The effect of interactions of *PPARG2* genotype and covariates on HbA1c were examined with a multiple regression model. The statistical test for an interaction was applied to a product term of a dichotomous *PPARG2* genotype and each covariate (ie, age, BMI, energy intake, alcohol consumption, and family history). On the basis of these 5 interaction tests the corrected significance threshold level, using the Bonferroni method, was *P* = 0.05/5 = 0.01. These analyses were stratified by sex because the distributions of clinical risk factors and mean HbA1c levels were significantly different between men and women.

Odds ratios (ORs) and 95% CIs of *PPARG2* genotype and the clinical risk factors for high-normal HbA1c (≥5.7% NGSP) were estimated using logistic regression models adjusted for potential confounders (age, BMI, energy intake, alcohol consumption, smoking, physical activity, and family history of diabetes). In this analysis we excluded 590 participants (332 men and 258 women) whose family history of diabetes was unknown. For further analysis, we excluded subjects who indicated that they had restricted their food intake due to their results on blood tests for glucose or cholesterol.

## RESULTS

The genotype frequency was within the Hardy–Weinberg equilibrium (93.6% in Pro12Pro, 6.3% in Pro12Ala, and 0.1% in Ala12Ala for expected values of 93.7%, 6.1%, and 0.1%, respectively).^11^ The background characteristics of the participants are summarized in Table 1. As compared with women, men had significantly higher values for HbA1c, BMI, energy intake, carbohydrate/energy intake, prevalence of current smokers, and prevalence of current drinkers. Women had higher fat/energy and protein/energy intakes. Age, physical activity level, and prevalence of a first-degree family history of diabetes were similar between sexes.

**Table 1.** Characteristics of study subjects by sex

	Men (*n* = 1280)	Women (*n* = 1356)	*P*_for difference_^a^
Serum HbA1c (%) NGSP	5.19 ± 0.63	5.12 ± 0.48	0.0033
Age (y)	57.3 ± 8.6	57.0 ± 8.4	0.3338
Body mass index (kg/m^2^)	23.7 ± 3.0	22.6 ± 3.1	<0.0001
Total energy intake (kcal/d)	1948.5 ± 355.3	1561 ± 258.7	<0.0001
Fat (energy %)	19.8 ± 5.1	25.7 ± 5.7	<0.0001
Protein (energy %)	11.8 ± 1.8	13.4 ± 1.9	<0.0001
Carbohydrate (energy %)	57.7 ± 6.3	55.7 ± 5.4	<0.0001
Physical activity level (MET·h)	13.4 ± 13.3	12.5 ± 11.6	0.0894
Current alcohol drinkers, *n* (%)	962 (75.2)	444 (32.7)	<0.0001
Current smoking, *n* (%)	364 (28.4)	86 (6.3)	<0.0001
Family history of diabetes, *n* (%)	188 (14.7)	215 (15.9)	0.8726

NGSP, National Glycohemoglobin Standardization Program; MET·h, metabolic equivalent-hours per day.

Variables are presented as mean ± SD for continuous variables and as number (%) for categorical variables.

^a^ *P* values for sex differences are based on the *t* test, for continuous variables, and the chi-square test, for categorical variables.

Tables 2a and 2b show the associations between HbA1c and clinical risk factors according to sex. Among men and women, the adjusted mean HbA1c was higher in older age groups and higher BMI categories and among participants with a first-degree family history of diabetes. In men, energy and alcohol intakes were inversely associated with adjusted mean HbA1c. In subsequent analysis that excluded subjects who had restricted their food intake due to their results on blood testing, the significant inverse association between energy and adjusted HbA1c in men disappeared (*P* for trend = 0.2786), whereas that between alcohol intake and adjusted HbA1c remained significant (*P* for trend = 0.0026). Carbohydrate intake was positively associated, and fat and alcohol intakes were inversely associated, with crude mean HbA1c in women; however, these associations disappeared after adjustment for possible confounders. No significant associations of protein intake, physical activity, or smoking with adjusted mean HbA1c were observed for either sex.

**Table 2a.** Crude and adjusted means^a^ (%) and 95% CIs of HbA1c (NGSP) by clinical risk factors and parental family history of diabetes in 1280 men

	Crude mean	(95% CI)	Adjusted mean^a^	(95% CI)
Age				
35–39	5.31	(5.12–5.50)	5.28	(5.09–5.46)
40–49	5.45	(5.36–5.54)	5.43	(5.35–5.52)
50–59	5.60	(5.54–5.66)	5.59	(5.53–5.65)
60–69	5.55	(5.49–5.60)	5.56	(5.51–5.61)
	*P*_for trend_ = 0.0316		*P*_for trend_ = 0.0002	
BMI (kg/m^2^)				
Q1: <21.7	5.45	(5.38–5.52)	5.44	(5.37–5.51)
Q2: 21.7 to <23.6	5.49	(5.42–5.56)	5.50	(5.43–5.56)
Q3: 23.6 to <25.4	5.58	(5.51–5.65)	5.56	(5.49–5.63)
Q4: ≥25.4	5.65	(5.58–5.72)	5.65	(5.59–5.72)
	*P*_for trend_ < 0.0001		*P*_for trend_ < 0.0001	
Energy (kcal/day)				
Q1: <1700	5.62	(5.55–5.70)	5.62	(5.54–5.69)
Q2: 1700 to <1880	5.53	(5.46–5.60)	5.52	(5.45–5.59)
Q3: 1880 to <2100	5.55	(5.48–5.62)	5.54	(5.48–5.61)
Q4: ≥2100	5.47	(5.41–5.54)	5.49	(5.42–5.56)
	*P*_for trend_ = 0.0070		*P*_for trend_ = 0.0332	
Fat (energy %)				
Q1: <16.6	5.53	(5.46–5.60)	5.55	(5.47–5.62)
Q2: 16.6 to <19.2	5.59	(5.52–5.66)	5.58	(5.51–5.65)
Q3: 19.2 to <22.6	5.51	(5.44–5.58)	5.51	(5.44–5.58)
Q4: ≥22.6	5.54	(5.47–5.61)	5.52	(5.45–5.59)
	*P*_for trend_ = 0.8066		*P*_for trend_ = 0.3559	
Protein (energy %)				
Q1: <10.6	5.51	(5.44–5.58)	5.55	(5.48–5.62)
Q2: 10.6 to <11.6	5.54	(5.47–5.61)	5.55	(5.48–5.62)
Q3: 11.6 to <12.6	5.55	(5.48–5.63)	5.54	(5.47–5.61)
Q4: ≥12.6	5.55	(5.48–5.62)	5.51	(5.44–5.58)
	*P*_for trend_ = 0.4108		*P*_for trend_ = 0.4339	
Carbohydrate (energy %)				
Q1: <54.2	5.51	(5.44–5.58)	5.52	(5.44–5.61)
Q2: 54.2 to <58.5	5.53	(5.46–5.60)	5.52	(5.45–5.59)
Q3: 58.5 to <61.9	5.51	(5.44–5.58)	5.50	(5.42–5.57)
Q4: ≥61.9	5.61	(5.54–5.68)	5.61	(5.53–5.70)
	*P*_for trend_ = 0.0904		*P*_for trend_ = 0.2278	
Physical activity level (MET·h/day)^b^				
Q1: <4.1	5.54	(5.47–5.61)	5.53	(5.46–5.60)
Q2: 4.1 to <9.1	5.53	(5.46–5.60)	5.52	(5.45–5.58)
Q3: 9.1 to <18.0	5.56	(5.49–5.63)	5.55	(5.48–5.62)
Q4: ≥18.0	5.54	(5.47–5.61)	5.56	(5.48–5.63)
	*P*_for trend_ = 0.9293		*P*_for trend_ = 0.5414	
Alcohol^b^				
Never	5.60	(5.53–5.68)	5.59	(5.52–5.67)
Former	5.57	(5.34–5.81)	5.55	(5.32–5.79)
Current 0.1–22.9 g/d	5.54	(5.48–5.60)	5.55	(5.49–5.61)
23.0–45.9 g/d	5.55	(5.47–5.63)	5.55	(5.48–5.63)
46.0+ g/d	5.44	(5.36–5.52)	5.44	(5.36–5.52)
	*P*_for trend_ = 0.0082		*P*_for trend_ = 0.0153	
Smoking				
Never	5.51	(5.44–5.58)	5.50	(5.43–5.57)
Former	5.54	(5.49–5.60)	5.54	(5.48–5.59)
Current 1–19 cigarettes/d	5.50	(5.38–5.61)	5.50	(5.39–5.62)
20–39 cigarettes/d	5.59	(5.50–5.67)	5.60	(5.52–5.69)
≥40 cigarettes/d	5.67	(5.46–5.88)	5.67	(5.46–5.88)
	*P*_for trend_ = 0.1338		*P*_for trend_ = 0.0508	
Family history of diabetes				
Positive	5.73	(5.64–5.82)	5.71	(5.62–5.80)
Negative	5.50	(5.46–5.55)	5.51	(5.47–5.55)
Unknown	5.52	(5.45–5.60)	5.51	(5.44–5.58)
	*P*_for trend_ < 0.0001		*P*_for trend_ < 0.0001	

NGSP, National Glycohemoglobin Standardization Program; MET·h, metabolic equivalent-hours per day.

^a^Adjusted for age (continuous), BMI (continuous), energy intake (continuous), physical activity level (continuous), ethanol intake (never, former, or current drinker consuming 0.1–22.9, 23.0–45.9, or ≥46 g ethanol/d), and smoking (never, former, or current smoker of 1–19, 20–39, or ≥40 cigarettes/d), and family history of diabetes (positive, negative, or unknown).

^b^Alcohol intake data were missing for 20 men. Physical activity data were missing for 9 men.

**Table 2b.** Crude and adjusted means^a^ (%) and 95% CIs of HbA1c (NGSP) by clinical risk factors and parental family history of diabetes in 1356 women

	Crude mean	(95% CI)	Adjusted mean^a^	(95% CI)
Age				
35–39	5.21	(5.08–5.33)	5.25	(5.13–5.38)
40–49	5.30	(5.23–5.36)	5.31	(5.24–5.37)
50–59	5.49	(5.45–5.53)	5.50	(5.45–5.54)
60–69	5.55	(5.51–5.58)	5.54	(5.50–5.58)
	*P*_for trend_ < 0.0001		*P*_for trend_ < 0.0001	
Body mass index (kg/m^2^)				
Q1: <20.5	5.39	(5.34–5.45)	5.43	(5.38–5.48)
Q2: 20.5 to <22.4	5.40	(5.35–5.45)	5.40	(5.35–5.45)
Q3: 22.4 to <24.3	5.51	(5.46–5.56)	5.50	(5.45–5.55)
Q4: ≥24.3	5.60	(5.55–5.65)	5.58	(5.53–5.63)
	*P*_for trend_ < 0.0001		*P*_for trend_ < 0.0001	
Energy (kcal/day)				
Q1: <1400	5.43	(5.38–5.49)	5.45	(5.39–5.50)
Q2: 1400 to <1540	5.51	(5.46–5.56)	5.51	(5.46–5.56)
Q3: 1540 to <1670	5.44	(5.39–5.49)	5.44	(5.39–5.49)
Q4: ≥1670	5.51	(5.46–5.56)	5.51	(5.46–5.56)
	*P*_for trend_ = 0.1957		*P*_for trend_ = 0.3222	
Fat (energy %)				
Q1: <21.9	5.51	(5.46–5.56)	5.48	(5.42–5.53)
Q2: 21.9 to <25.3	5.52	(5.47–5.57)	5.52	(5.47–5.57)
Q3: 25.3 to <28.8	5.45	(5.39–5.50)	5.45	(5.40–5.50)
Q4: ≥28.8	5.43	(5.38–5.48)	5.46	(5.41–5.52)
	*P*_for trend_ = 0.0101		*P*_for trend_ = 0.3459	
Protein (energy %)				
Q1: <12.1	5.47	(5.41–5.52)	5.49	(5.44–5.54)
Q2: 12.1 to <13.2	5.47	(5.42–5.52)	5.47	(5.42–5.52)
Q3: 13.2 to <14.3	5.48	(5.43–5.54)	5.48	(5.43–5.53)
Q4: ≥14.3	5.49	(5.43–5.54)	5.47	(5.42–5.52)
	*P*_for trend_ = 0.5146		*P*_for trend_ = 0.7937	
Carbohydrate (energy %)				
Q1: <53.2	5.40	(5.35–5.45)	5.45	(5.40–5.51)
Q2: 53.2 to <56.3	5.48	(5.42–5.53)	5.49	(5.44–5.55)
Q3: 56.3 to <59.1	5.49	(5.44–5.54)	5.46	(5.41–5.51)
Q4: ≥59.1	5.54	(5.49–5.59)	5.50	(5.45–5.56)
	*P*_for trend_ = 0.0002		*P*_for trend_ = 0.3479	
Physical activity level (MET·h/day)^b^				
Q1: <4.6	5.42	(5.37–5.47)	5.44	(5.39–5.49)
Q2: 4.6 to <9.3	5.51	(5.46–5.56)	5.51	(5.46–5.56)
Q3: 9.3 to <17.4	5.48	(5.43–5.53)	5.48	(5.43–5.53)
Q4: ≥17.4	5.49	(5.44–5.54)	5.48	(5.43–5.53)
	*P*_for trend_ = 0.1421		*P*_for trend_ = 0.3859	
Alcohol^b^				
Never	5.50	(5.47–5.53)	5.49	(5.46–5.52)
Former	5.40	(5.21–5.60)	5.40	(5.21–5.59)
Current 0.1–22.9 g/d	5.44	(5.39–5.49)	5.46	(5.41–5.50)
23.0–45.9 g/d	5.49	(5.33–5.65)	5.53	(5.37–5.68)
46.0+ g/d	5.25	(5.02–5.47)	5.25	(5.03–5.47)
	*P*_for trend_ = 0.0108		*P*_for trend_ = 0.1361	
Smoking				
Never	5.49	(5.46–5.51)	5.48	(5.45–5.51)
Former	5.39	(5.27–5.52)	5.44	(5.32–5.57)
Current 1–19 cigarettes/d	5.32	(5.19–5.45)	5.39	(5.26–5.52)
20–39 cigarettes/d	5.49	(5.32–5.66)	5.54	(5.37–5.71)
≥40 cigarettes/d				
	*P*_for trend_ = 0.0760		*P*_for trend_ = 0.5833	
Family history of diabetes				
Positive	5.60	(5.53–5.66)	5.61	(5.55–5.67)
Negative	5.45	(5.42–5.48)	5.46	(5.43–5.49)
Unknown	5.45	(5.39–5.52)	5.42	(5.36–5.48)
	*P*_for trend_ < 0.0001		*P*_for trend_ < 0.0001	

NGSP, National Glycohemoglobin Standardization Program; MET·h, metabolic equivalent-hours per day.

^a^Adjusted for age (continuous), body mass index (continuous), energy intake (continuous), physical activity level (continuous), ethanol intake (never, former, or current drinker consuming 0.1–22.9, 23.0–45.9, or ≥46 g ethanol/d), and smoking (never, former, or current smoker of 1–19, 20–39, or ≥40 cigarettes/d), and family history of diabetes (positive, negative, or unknown).

^b^Alcohol intake data were missing for 22 women. Physical activity data were missing for 14 women.

Table 3 shows associations between HbA1c and *PPARG2* genotypes. The adjusted mean HbA1c was lower in female but not male Ala allele carriers. However, there was no significant interaction between sex and *PPARG2* allele on HbA1c.

**Table 3.** Crude and adjusted mean HbA1c by PPAR2 gene polymorphism genotype and sex

	Men (*n* = 1280)					Women (*n* = 1356)				
		
	*n*	Mean^a^	(95% CI)	Mean^b^	(95% CI)	*n*	Mean^a^	(95% CI)	Mean^b^	(95% CI)
*PPARG2* (rs1801282) genotype										
Pro12Pro	1194	5.54	(5.50–5.57)	5.54	(5.50–5.57)	1274	5.48	(5.46–5.51)	5.48	(5.46–5.51)
Pro12Ala	84	5.60	(5.41–5.78)	5.55	(5.42–5.69)	82	5.36	(5.28–5.45)	5.38	(5.27–5.48)
Ala12Ala	2	5.40	(4.75–6.05)	5.40	(4.52–6.27)	0				
		*P* = 0.6947		*P* = 0.9070			*P* = 0.0324		*P* = 0.0493	

^a^Crude means.

^b^Adjusted for age (continuous), body mass index (continuous), energy intake (continuous), physical activity (continuous), ethanol intake (never, former, or current drinker consuming 0.1–22.9, 23.0–45.9, or ≥46 g ethanol/d), and smoking (never, former, or current smoker of 1–19, 20–39, or ≥40 cigarettes/d), and family history of diabetes (positive, negative, or unknown).

The associations between HbA1c and important clinical risk factors (Tables 2a and 2b) were examined by *PPARG2* genotype and sex (Table 4). Positive associations of age, BMI, and family history with HbA1c were seen among participants with the Pro12Pro genotype but not among Ala allele carriers. In addition, a significant interaction between BMI and *PPARG2* genotype on HbA1c was observed in women. However, this interaction was not statistically significant after Bonferroni correction. Energy and alcohol intakes were not significantly associated with HbA1c in analysis stratified by genotype or sex.

**Table 4.** Adjusted mean^a^ (%) HbA1c by age, BMI, and parental family history of diabetes according to *PPARG2* genotype in men and women

	Men (*n* = 1280)		*P* for difference	*P* for interaction	Women (*n* = 1356)		*P* for difference	*P* for interaction
		
	*PP* (*n* = 1194)	*PA* + *AA* (*n* = 86)			*PP* (*n* = 1274)	*PA* + *AA* (*n* = 82)		
Age								
35–49	5.43 (5.38–5.48)	5.40 (5.17–5.62)	0.7846	0.7254	5.28 (5.24–5.32)	5.24 (5.11–5.34)	0.5152	0.5552
50–69	5.56 (5.52–5.61)	5.59 (5.43–5.75)	0.7717		5.53 (5.50–5.56)	5.41 (4.93–5.19)	0.0811	
	*P* = 0.0003	*P* = 0.5388			*P* < 0.0001	*P* = 0.1413		
BMI								
Q1 + Q2	5.46 (5.41–5.50)	5.50 (5.34–5.67)	0.5870	0.7500	5.40 (5.37–5.43)	5.43 (5.30–5.55)	0.6232	0.0286
Q3 + Q4	5.62 (5.56–5.68)	5.60 (5.38–5.82)	0.8779		5.57 (5.53–5.61)	5.37 (5.20–5.54)	0.0222	
	*P* < 0.0001	*P* = 0.6439			*P* < 0.0001	*P* = 0.2842		
Energy								
Q1 + Q2	5.56 (5.50–5.62)	5.70 (5.49–5.92)	0.2041	0.0673	5.48 (5.45–5.52)	5.34 (5.19–5.50)	0.0764	0.3936
Q3 + Q4	5.52 (5.48–5.56)	5.39 (5.23–5.55)	0.1407		5.48 (5.44–5.52)	5.43 (5.28–5.57)	0.4847	
	*P* = 0.3685	*P* = 0.0678			*P* = 0.6663	*P* = 0.4948		
Alcohol								
Never + Former	5.60 (5.52–5.67)	5.66 (5.38–5.95)	0.6557	0.7657	5.50 (5.47–5.53)	5.44 (4.96–5.22)	0.3807	0.3695
Current	5.52 (5.48–5.56)	5.52 (5.36–5.67)	0.9605		5.44 (5.40–5.49)	5.32 (5.15–5.49)	0.1774	
	*P* = 0.1324	*P* = 0.8174			*P* = 0.3574	*P* = 0.3742		
Family history of diabetes^b^								
Positive	5.73 (5.64–5.81)	5.50 (5.13–5.87)	0.2290	0.5604	5.60 (5.50–5.69)	5.59 (5.02–6.15)	0.9651	0.7307
Negative	5.51 (5.46–5.55)	5.45 (5.29–5.62)	0.5411		5.46 (5.43–5.49)	5.37 (5.26–5.47)	0.0983	
Unknown	5.50 (5.42–5.58)	5.89 (5.59–6.18)	0.0133		5.46 (5.40–5.52)	5.35 (5.14–5.56)	0.3151	
	*P* < 0.0001	*P* = 0.5172			*P* = 0.0002	*P* = 0.1876		

^a^Adjusted for age (continuous), body mass index (BMI; continuous), energy intake (continuous), physical activity level (continuous), ethanol intake (never, former, or current drinker consuming 0.1–22.9, 23.0–45.9, or ≥46 g ethanol/d), and smoking (never, former, or current smoker of 1–19, 20–39, or ≥40 cigarettes/d), and family history of diabetes (positive, negative, or unknown).

^b^ *P*_for difference_ and *P*_for interaction_ were compared using the adjusted means of individuals with and without a family history of diabetes.

The effects of *PPARG2* genotype and clinical risk factors on the risk of a high-normal HbA1c (≥5.7% NGSP) are shown in Table 5. Older age, higher BMI, and a family history of diabetes were associated with significantly higher ORs for a high-normal HbA1c. Women and Ala allele carriers had lower ORs for a high-normal HbA1c, though these ORs were not statistically significant. Concerning energy intake, a significant inverse trend with high-normal HbA1c was observed even after adjusting for possible confounders (Model 2), but this disappeared after excluding subjects who had restricted their food intake due to the results of blood examinations (Model 3). Concerning alcohol intake, a significant inverse trend with high-normal HbA1c was observed in the crude model (Model 1); however, the association disappeared after adjusting for possible confounders (Model 2).

**Table 5.** Odds ratios (ORs) and 95% CIs for high HbA1c (≥5.7 NGSP) according to genotype and clinical risk factors among 2046 men and women

	*n*	High HbA1c	Model 1		Model 2		Model 3	
			
			OR	95% CI	OR	95% CI	OR	95% CI
Sex								
Men	948	231	1.00	(reference)	1.00	(reference)	1.00	(reference)
Women	1098	243	0.88	(0.72–1.08)	0.92	(0.69–1.25)	1.05	(0.74–1.49)

*PPARG* Pro12Ala (C/G) (rs1801282)								
PP	1921	454	1.00	(reference)	1.00	(reference)	1.00	(reference)
PA + AA	125	20	0.62	(0.38–1.00)	0.61	(0.36–1.03)	0.54	(0.28–1.04)
								
Age								
35–39	86	5	1.00	(reference)	1.00	(reference)	1.00	(reference)
40–49	347	47	2.54	(0.98–6.59)	2.00	(0.76–5.28)	2.18	(0.74–6.44)
50–59	760	187	5.29	(2.11–13.24)	4.21	(1.65–10.75)	4.29	(1.51–12.19)
60–69	853	235	6.16	(2.47–15.39)	4.61	(1.81–11.78)	4.31	(1.51–12.29)
			*P*_for trend_ < 0.0001		*P*_for trend_ < 0.0001		*P*_for trend_ = 0.0002	
BMI								
Q1 (lowest)	523	72	1.00	(reference)	1.00	(reference)	1.00	(reference)
Q2	515	98	1.47	(1.06–2.05)	1.34	(0.95–1.90)	1.24	(0.84–1.83)
Q3	513	137	2.28	(1.66–3.13)	2.04	(1.46–2.85)	1.88	(1.28–2.75)
Q4 (highest)	494	137	3.20	(2.34–4.34)	2.87	(2.07–3.98)	2.56	(1.75–3.75)
			*P*_for trend_ < 0.0001		*P*_for trend_ < 0.0001		*P*_for trend_ < 0.0001	
Energy (kcal/day)								
Q1 (lowest)	431	105	1.00	(reference)	1.00	(reference)	1.00	(reference)
Q2	493	126	1.07	(0.79–1.44)	0.95	(0.68–1.33)	0.98	(0.66–1.47)
Q3	532	117	0.88	(0.65–1.18)	0.74	(0.53–1.03)	0.62	(0.41–0.94)
Q4 (highest)	590	126	0.84	(0.63–1.13)	0.76	(0.54–1.06)	0.81	(0.54–1.22)
			*P*_for trend_ = 0.1221		*P*_for trend_ = 0.0407		*P*_for trend_ = 0.1350	
Alcohol^a^								
Never	885	234	1.00	(reference)	1.00	(reference)	1.00	(reference)
Former	43	5	0.37	(0.14–0.94)	0.35	(0.13–0.93)	0.30	(0.09–1.02)
Current 0.1–22.9 g/d	675	139	0.72	(0.57–0.92)	0.77	(0.58–1.00)	0.93	(0.68–1.28)
23.0–45.9 g/d	220	59	1.02	(0.73–1.42)	1.13	(0.74–1.75)	1.52	(0.91–2.55)
46.0+ g/d	192	33	0.58	(0.39–0.86)	0.65	(0.37–1.16)	1.12	(0.57–2.21)
			*P*_for trend_ = 0.0120		*P*_for trend_ = 0.1583		*P*_for trend_ = 0.6605	
Family history of diabetes								
Negative	1668	344	1.00	(reference)	1.00	(reference)	1.00	(reference)
Positive	378	130	2.02	(1.58–2.57)	2.15	(1.66–2.80)	2.15	(1.57–2.94)

Model 1: Crude OR.

Model 2: Adjusted for *PPARG2* genotype (PP or PA + AA), age (continuous), body mass index (BMI; continuous), energy intake (continuous), physical activity (continuous), ethanol intake (never, former, or current drinker consuming 0.1–22.9, 23.0–45.9, or ≥46 g ethanol/d), and smoking (never, former, or current smoker of 1–19, 20–39, or ≥40 cigarettes/d), and family history of diabetes (positive or negative).

Model 3: Further excluded subjects who answered that they had restricted food intake due to the results of blood testing for glucose and cholesterol. Ajusted for same variables in Model 2.

^a^Alcohol intake data were missing for 31 subjects.

## DISCUSSION

It is assumed that genetic factors modify the effects of known risk factors for diabetes. *PPARG2* is a ligand-activated transcription factor belonging to the nuclear hormone receptor superfamily,^18^ and it regulates various genes involved in lipid and glucose metabolism. *PPARG2* deficiency is believed to improve insulin resistance by decreasing muscle/liver triglyceride content and preventing adipocyte hypertrophy.^19^ Thus, the effects of lifestyle-related factors on HbA1c can be modified by the Pro12Ala genotype.

Our large cross-sectional study found significant positive associations of HbA1c with age, BMI, and family history of diabetes in men and women with the Pro12Pro genotype but not in Ala allele carriers. This appears to support the hypothesis that Ala allele carriers are less affected by the clinical risk factors of diabetes and that the Ala allele protects against diabetes development.^7^ However, the analysis of Ala allele carriers was underpowered due to the small sample size and failed to detect clinical risk among this subpopulation. Similarly, a significant association of this polymorphism with high-normal HbA1c was not detected, also due to the low prevalence of Ala allele carriers. The OR for high-normal HbA1c associated with this polymorphism was lower than those associated with age, BMI, and family history. Thus, the impact of this polymorphism on diabetes development is probably lesser than the effects of known clinical risk factors. Family history can be considered a surrogate for other genetic factors, as well as for family-related clinical risk factors. In addition, family history is regarded as more appropriate than genotype for prediction of individual risk. Lyssenko et al examined if genetic factors were better than established clinical risk factors at predicting progression to diabetes and found that 11 common genetic variants associated with diabetes risk had smaller effects than family history on the ability to predict diabetes development.^6^

The protective effect of the Ala allele was evident in men and women with higher BMIs, and a significant interaction between Pro12Ala genotype and BMI on HbA1c was observed in women. However, this significant interaction disappeared after Bonferroni correction. Several studies found that insulin sensitivity was higher in overweight or obese people with the Ala allele than in those without it.^20^^–^^24^ The few studies investigating potential obesity–genotype interactions^22^^,^^23^^,^^25^ found significant^22^ and borderline-significant^23^ interactions. To the best of our knowledge, our study is the first to examine the interaction between Pro12Ala genotype and BMI on HbA1c in an Asian general population, which has a lower proportion of extremely obese persons as compared with white populations. It is plausible that the Ala allele protects against an increase in HbA1c that would normally arise in obese people, because having the allele prevents adipocyte hypertrophy and insulin resistance. We speculate that the lower frequency of Ala allele carriers among Japanese as compared with whites^7^ may partially explain why the prevalence of type 2 diabetes in the Japanese population is not dramatically lower than in Western people, despite the lower prevalence of obesity in Japan.^3^^,^^26^

We found that the Ala allele had a significant protective effect against increased HbA1c in women but not in men and that there were no interactions by sex or genotype. Concerning this sex difference, 2 previous studies reported relationships between the Ala allele and insulin resistance by sex.^21^^,^^27^ One Italian study found no association between the Pro12Ala genotype and insulin sensitivity in either sex.^27^ However, a Spanish cross-sectional study reported associations of Ala carriers with lower fasting insulin and higher insulin sensitivity among women only.^21^ The authors of that study speculated that the different fat distributions of men and women modified the associations. Women have more subcutaneous (ie, white) adipose tissue. It is suspected that carrying the Ala allele prevents adipocyte hypertrophy in white adipose tissue, thereby decreasing the molecules that cause insulin resistance.^19^ Hormonal factors potentially modulate this sex-specific association. *PPARG2* activation has been reported to inhibit aromatase, a key enzyme in the conversion of androgens to estrogens.^28^ In addition, the possibility of sex-specific lifestyle effects on HbA1c should be considered.^29^ In men, significantly higher levels of BMI, energy intake, and cigarette smoking might have greater effects than genotype on HbA1c.

In this study, we observed a favorable effect of alcohol intake on HbA1c in men but not in women. This favorable effect of alcohol intake against high-normal HbA1c was not clear after adjustment for possible confounding factors. A meta-analysis of prospective observational studies noted that moderate alcohol consumption lowered the risk of type 2 diabetes^30^; however, results from Japanese studies have been inconsistent.^31^ Follow-up of our study is needed to provide further information on the Japanese population.

Our study has several methodological issues that warrant discussion. First, we used HbA1c instead of fasting glucose and insulin to evaluate diabetes risk. However, HbA1c is more commonly used to diagnose diabetes and can be used to identify individuals at higher risk of developing diabetes.^8^ It has been reported that high-normal HbA1c is a strong predictor of type 2 diabetes in the Japanese population.^9^^,^^32^ We were aware that excluding 1768 participants with missing HbA1c data might cause selection bias. However, we found that the proportions of *PPARG2* genotypes among the excluded data were not different from those in the analyzed data. A second limitation of this research is that we did not standardize HbA1c measurements among the laboratories in this study. This is a possible cause for the neutral results regarding the association between HbA1c and *PPARG2* genotype. Third, the cross-sectional nature of our study limits our ability to determine causation, even though we excluded participants who were receiving medication for type 2 diabetes. Fourth, there may be intrinsic information bias in our assessments of lifestyle-related factors, dietary factors, and family history. If present, however, any misclassification would be nondifferential with respect to *PPARG2* genotype and would likely underestimate the true associations. Finally, residual confounding by known and unknown risk factors may be present, although we adjusted for potential confounding factors in multivariate analysis.

In conclusion, the *PPARG2* Pro12Ala polymorphism might modify the risk factors of diabetes. The impact of this allele in the Japanese population appears to be lower than the effects of age, BMI, and family history. These findings highlight the importance of known risk factors, versus genetic polymorphism, in common diseases.

## ONLINE ONLY MATERIALS

The Japanese-language abstract for articles can be accessed by clicking on the tab labeled Supplementary materials at the journal website http://dx.doi.org/10.2188/jea.JE20120078.

Abstract in Japanese.
