# Supplementary material for: Effect of the PPARG2 Pro12Ala Polymorphism and Clinical Risk Factors for Diabetes Mellitus on HbA1c in the Japanese General Population
Source: J Epidemiol. 2012 Nov 5;22(6):523–31. doi: 10.2188/jea.JE20120078 (PMC3798564; doi:10.2188/jea.JE20120078)
Supplement: Abstract in Japanese. [file je-22-523-s001.pdf]

PPARG2 遺伝子の Pro12Ala 多型と HbA1c との関連

原めぐみ[1]、檜垣靖樹[2]、田口尚人[1]、新地浩一[3]、森田えみ[4]、内藤真理子[4]、浜島信之[4]、高嶋直敬[5]、鈴木貞夫[6]、中村昭彦[7]、大中桂三[8]、上村浩一[9]、西田秀樹[10]、細野覚代[11]、三上春夫[12]、久保充明[13]、田中英夫[11]

[1]佐賀大学医学部社会医学講座予防医学分野、[2]福岡大学スポーツ科学部運動生理学研究室、[3]佐賀大学大学院医学系研究科地域・国際保健看護学講座、[4]名古屋大学大学院医学系研究科予防医学、[5]滋賀医科大学社会医学講座公衆衛生学、[6]名古屋市立大学大学院医学研究科公衆衛生学分野、[7]鹿児島大学大学院医歯学総合研究科国際島嶼医療学講座、[8]九州大学大学院医学研究院老年医学分野、[9]徳島大学大学院ヘルスバイオサイエンス研究部予防医学、[10]京都府立医科大学大学院医学研究科地域保健医療疫学、[11]愛知県がんセンター研究所疫学・予防部、[12]千葉県がんセンターがん登録・予防疫学研究室、[13]理科学研究所ゲノム医科学研究センター

背景：肥満や脂質代謝に関連する遺伝子の *PPARG2* の変異型の人で糖尿病のリスクが低いことが分かってきたが、糖尿病の危険因子と *PPARG2* の遺伝子型が HbA1c に与える影響は十分に検証されていない。そこで、*PPARG2* の遺伝子の型と糖尿病の危険因子が HbA1c 値に及ぼす影響、および交互作用について検討した。方法：J-MICC Study のベースライン調査に参加した 40～69 歳の男性 1281 人、女性 1356 人のデータを用いて横断研究を実施した。遺伝子多型は Multiplex PCR-based Invader assay 方法により決定した。男女別に、Pro12Ala 多型と HbA1c の関連について、開始時の年齢、エネルギー摂取量、身体活動量、飲酒状況、喫煙状況、BMI で調整し、共分散分析、重回帰分析を行った。結果：女性では、*PPARG2* 遺伝子の Pro12Ala 型及び Ala12Ala 型を持つ者は Pro12Pro 型を持つ者と比較して、HbA1c が有意に低かった。*PPARG2* が Pro/Pro 型の人は、加齢や肥満、家族歴があると HbA1c の上昇がみられたが、Pro/Ala または Ala/Ala 型の人ではみられなかった。女性では HbA1c 値に対し BMI と遺伝子多型による有意な交互作用がみられた。加齢や肥満、家族歴は HbA1c 高値と有意な関連がみられたが、*PPARG2* 遺伝子の多型と HbA1c 高値の間に有意な関連はみられなかった。結論：*PPARG2* 遺伝子の Pro12Ala 多型は、HbA1c と既知の危険因子の関連に影響を与える可能性があるが、一般集団において HbA1c 高値に対する影響は既知の危険因子に比べて小さかった。

キーワード：peroxisome proliferator-activated receptor-γ2、遺伝子多型、HbA1c、交互作用
